# A systematic review of training programmes for recruiters to randomised clinical trials

**DOI:** 10.1186/1745-6215-16-S2-P117

**Published:** 2015-11-16

**Authors:** Daisy Towsend, Nicola Mills, Jelena Savović, Jenny Donovan

**Affiliations:** 1 University of Bristol, Bristol, UK

## Background

Clinician related factors have been implicated as important reasons for low rates of recruitment to randomised controlled trials (RCTs). Clinicians can experience discomfort with some underlying principles of RCTs and experience difficulties in conveying them positively to potential trial participants. Recruiter training has been suggested to address identified problems but a synthesis of this research is lacking. We therefore systematically reviewed the available evidence on training interventions for RCT recruiters.

## Method

Studies that evaluated training programmes for trial recruiters were included. Those that provided only general communication training not linked to RCT recruitment were excluded. Data extraction and quality assessment were completed by two reviewers independently.

## Results

15 studies from 7,337 potentially eligible titles and abstracts were included in the review: three RCTs, three non-randomised controlled studies, six pre-test-post-test studies, two qualitative studies and a post-training questionnaire survey. Most studies were of moderate or weak quality. Training programmes were mostly set within cancer trials, and usually consisted of workshops with a mix of health professionals over one or two consecutive days covering generic and trial specific issues. Recruiter training programmes were well received and there was evidence that some increased recruiters’ self-confidence in communicating key RCT concepts. There was, however, very little evidence that this training increased recruitment rates or patient understanding, satisfaction, or levels of informed consent

## Conclusion

There is a need to develop training programmes that can enable recruiters to explain key concepts effectively and evaluate whether they lead to improved recruitment and informed consent in RCTs.

